# A rare case of colon obstruction due to gastrointestinal basidiobolomycosis in a 36-year-old woman

**DOI:** 10.1016/j.ijscr.2020.11.054

**Published:** 2020-11-24

**Authors:** Alaa Eid Aljohani, Bandar Alshemesi, Abdullatif Alshubaisheri, Abdulmajeed Alkraidis, Ali Alzahrani, Rami Sairafi

**Affiliations:** General Surgery Department, Security Forces Hospital, Riyadh, Saudi Arabia

**Keywords:** Basidiobolomycosis, Gastrointestinal basidiobolomycosis, Entomophthorales, *Basidiobolus ranarum*, Case report

## Abstract

**Introduction:**

Gastrointestinal basidiobolomycosis (GIB) is an uncommon fungal infection caused by*Basidiobolus ranarum*, with less than 80 cases reported in the literature.

**Presentation of case:**

A 36-year-old woman presented with colicky upper abdominal pain for 2 months and inability to pass stool for 2 days. Computed tomography of the abdomen and colonoscopy both showed a mass in the transverse colon. Colonoscopic biopsy suggested a zygomycosis. Laparotomy revealed a perforated mass in the transverse colon; the mass also involved the small bowel and stomach. En bloc resection was performed, and the incision was closed for a second-look procedure. The patient was admitted to the intensive care unit and placed on a ventilator and inotropes; however, her condition deteriorated, and she died on day 24 post admission. The histopathology report (obtained after the patient’s death) was consistent with GIB.

**Discussion:**

Gastrointestinal basidiobolomycosis presents with nonspecific signs and symptoms and so the diagnosis is easily missed or delayed. Timely, accurate diagnosis is crucially important, especially when there is intestinal obstruction and sepsis. Persistent severe abdominal pain in a patient with neutropenia should alert the physician to the possibility of a fungal infection. There are reports from several countries of basidiobolomycosis in immunocompetent hosts. The disease shows no age predilection, but males may be more susceptible. Treatment is with surgical debridement and antifungal drugs—ideally the lipid formulation of amphotericin B.

**Conclusion:**

Gastrointestinal basidiobolomycosis is a rare infection that can be fatal if not diagnosed and treated early.

## Highlights

• Colon Obstruction Due to Gastrointestinal Basidiobolomycosis is extremely rare.
• A fungal vascular invasion with tumor affecting multiple organ may present a challenging clinical situation.
• Early surgical resection remains the treatment of choice and plays a potentially curative role.

## 1 Introduction

Gastrointestinal basidiobolomycosis (GIB) is a rare emerging infection with a high mortality rate [1]. It can affect immunocompetent individuals. More research is needed to identify the factors that predispose to GIB. Because of the rarity of the disease, the diagnosis is often not suspected initially. We present a case of GIB in a 36-year-old immunocompetent woman.

## 2 Case presentation

A 36-year-old woman presented to the emergency department with complaints of upper abdominal pain for 2 months and inability to pass stool for 2 days. The pain was epigastric, colicky in nature, increasing in intensity, and radiating to the back. It was partially relieved by analgesics, but otherwise there were no alleviating or aggravating factors. There was associated nausea and vomiting (food particles), but no hematemesis. The patient also complained of recurrent attacks of night sweats, fever, chills, and fatigue, and weight loss of more than 11 kg over the previous 2 months. There was no history of chest pain, dyspnea, heart burn, reflux symptoms, dizziness, headache, or jaundice. At 17 years of age, the patient had been diagnosed with *Helicobacter pylori* infection at K.F.S.H. The infection had persisted despite several courses of treatment, and she was finally discharged on ranitidine. Her past history was also remarkable for recurrent benign nasal tumor (myxoma).

The patient was not on any regular medication. She had no known drug or food allergies. There was no family history of any gastrointestinal disease or malignancy, and no documented genetic disease.

On examination, the patient was conscious, alert, oriented, and mildly dehydrated. She looked pale and was obviously in pain. Blood pressure was 119/78 mmHg; heart rate, 110 beats per minute; temperature, 37.3 °C; and peripheral capillary oxygen saturation, 100 % on room air. The abdomen was soft, with no distention. A mass was palpable in the left upper quadrant; it measured about 16 × 10 cm in size and was dull on percussion. Bowel sounds were increased. Rectal examination was normal.

**Fig. 1:**
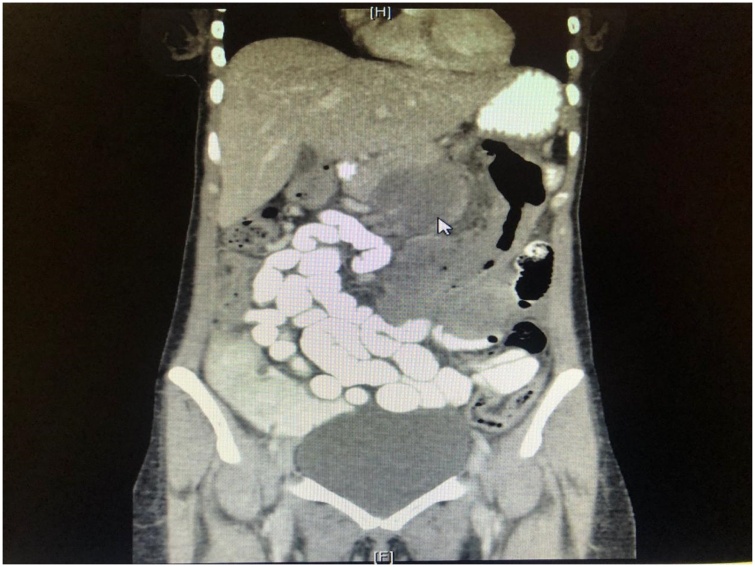
Coronal enhanced computed tomography scan of the abdomen showing an obstructing transverse colon mass (arrow).

**Fig. 2:**
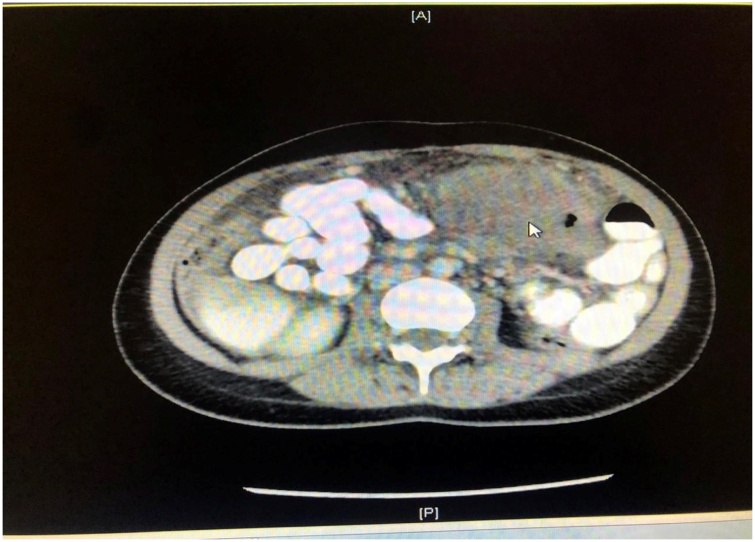
Axial enhanced computed tomography scan of the abdomen showing an obstructing transverse colon mass (arrow).

**Fig. 3:**
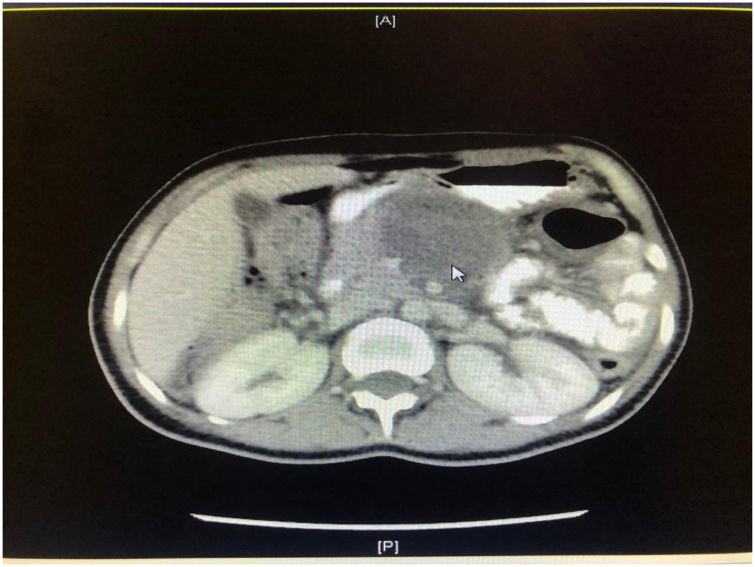
Axial enhanced computed tomography scan of the abdomen showing an obstructing transverse colon mass (arrow).

The laboratory results were as follows: white blood cell count, 17.5 × 10^9^/L, with 67.8 % neutrophils; hemoglobin, 12.5 g/dL; hematocrit, 38.1 %; platelet count, 328 × 10^9^/L; C-reactive protein, 320 mg/L; and serum lactate, 1.3 mmol/L. Kidney function, liver function, albumin 30, and serum electrolytes were within normal limits. Hepatitis profile and HIV test were negative. The coagulation profile was normal, and sepsis workup was negative. The chest radiograph was unremarkable. Abdominal computed tomography (CT) showed significant circumferential wall thickening of the transverse colon (extending about 16 cm), but no stricture; there was pericolic fat stranding. CT also revealed a necrotic lymph node, measuring 6 × 6 cm, lying posterior to the pancreatic head and displacing it anteriorly. Multiple enlarged mesenteric lymph nodes were also present, the largest measuring 1.2 cm in diameter (Fig. 1, Fig. 2, Fig. 3).

Based on these findings, out provisional diagnosis was an invading colonic mass; the differential diagnoses included lymphoma, colon cancer, and pancreatic cancer. The patient was admitted for further investigations. After multidisciplinary consultation, lymphoma involving the colon was considered the most likely possibility. Intravenous antibiotic was started and the patient was prepared for colonoscopy (with enema and Movicol). Colonoscopy revealed an ulcerated, fungating mass in the transverse colon, approximately 70 cm from the anal verge. There was narrowing of the lumen. Eight biopsy samples were obtained from the mass. Stenting not done because there was no definite obstruction. Following the colonoscopy, the patient started passing stool and tolerating oral fluid. However, at day 7 post admission, the patient started complaining of abdominal distention and inability to pass stool or flatus, Her vital signs were within normal limits, and abdominal examination findings were the same as before. She improved with conservative management. On day 12, colonoscopy biopsy result revealed zygomycosis.

The infectious disease specialist advised intravenous liposomal amphotericin B (300 mg daily for 3–6 months) and, if possible, surgical excision of the colonic mass plus the retroperitoneal lymph nodes. Soon after the first dose of amphotericin, the patient developed chills and tachycardia, and her Glasgow Coma Scale (GCS) score dropped to 7. The pupils were dilated and reactive. She also developed generalized edema. Suspecting anaphylaxis, we transferred the patient to the intensive care unit, where she was intubated. At admission to the ICU, her arterial blood gas parameters were as follows: pH, 7.51; PCO_2_, 27; HCO_3_ 23; and base deficit, −0.2. Blood examination showed the following: white blood cell count, 9 × 10^9^/L, with 74 % neutrophils, 17 % lymphocytes, and 6% eosinophils; hemoglobin 9 g/dL; hematocrit, 31.5 %; platelet count, 435 × 10^9^/L; erythrocyte sedimentation rate, 82 mm, serum lactate, 7 mmol/L; and C-reactive protein, 158 mg/L.

Intravenous ceftazidime was started in the ICU. The neurologist ordered brain CT and magnetic resonance imaging (MRI) to check the cause of the decreased level of consciousness. The otolaryngology team asked for sinus CT to exclude nasal zygomycosis. Brain CT showed a slight narrowing of the ventricular system with minimal loss of surrounding sulci. MRI showed bilateral thalamic ischemia with patent cerebral vessels, indicating total circulatory collapse, global hypoperfusion, and septic shock. CT of the paranasal sinuses showed bone resorption in the floor of the nasal cavity (hard palate), extending up to the pterygoid plate. There was a soft tissue density suggestive of a nasal tumor or postoperative change. Mucosal thickening in the left maxillary sinus was present. The patient’s family refused biopsy of the nasal mass.

On day 14, she developed sinus tachycardia (180 per minute) and fever. Echocardiogram was normal. Amphotericin B was resumed preceded by hydrocortisone. Surgical intervention was not offered at the time as we hoped that reduction in mass size would allow complete resection later. Over the next week, her condition improved. GCS score increased to 14/15, and she could be extubated. She resumed oral fluids, but started vomiting again. An interventional radiologist inserted a nasojejunal tube to bypass the compressed area of the stomach and duodenum.

**Fig. 4:**
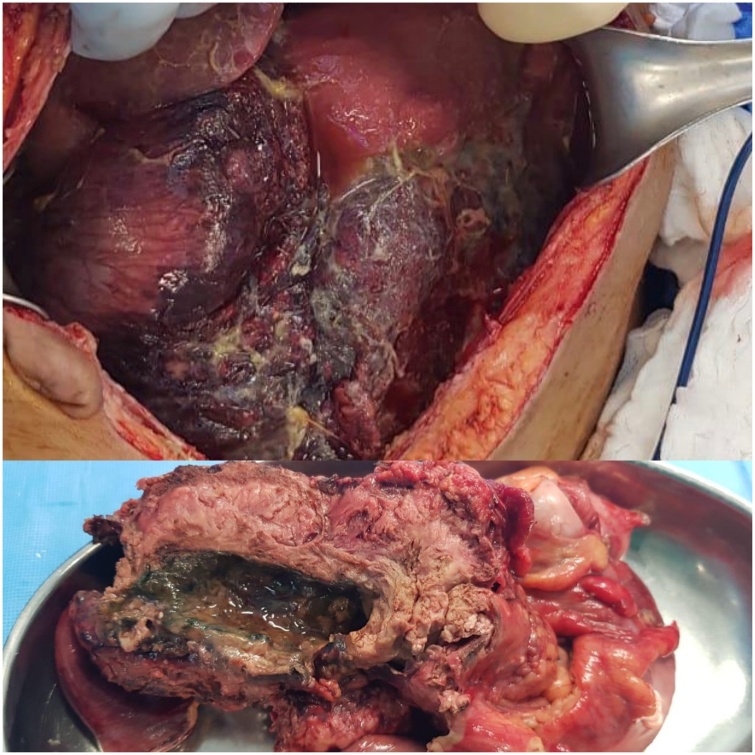
En bloc resected specimen showing transverse colon wall thickening with necrotic debris in the lumen.

On day 21, her condition deteriorated. She developed hypotension, tachycardia, and respiratory acidosis. She was intubated again. Her abdomen was tensely distended and silent. The serum lactate level was 19 mmol/L. CT abdomen showed air under the diaphragm. At emergency laparotomy, a perforated mass was found in the transverse colon. The mass involved the transverse colon, small bowel, and stomach. The small bowel was ischemic from the duodenojejunal junction up to the distal jejunum. However, there was viable bowel 120 cm from the ileocecal valve. En bloc excision was performed (Fig. 4), and the abdominal incision was closed for a second-look procedure (damage control surgery). Abdominal fluid was sent for cytology. The patient was returned to the ICU and placed on ventilator and inotropic support; however, her condition continued to deteriorate and she died on day 24 post admission. The second-look surgery could not be performed before her death.

**Fig. 5:**
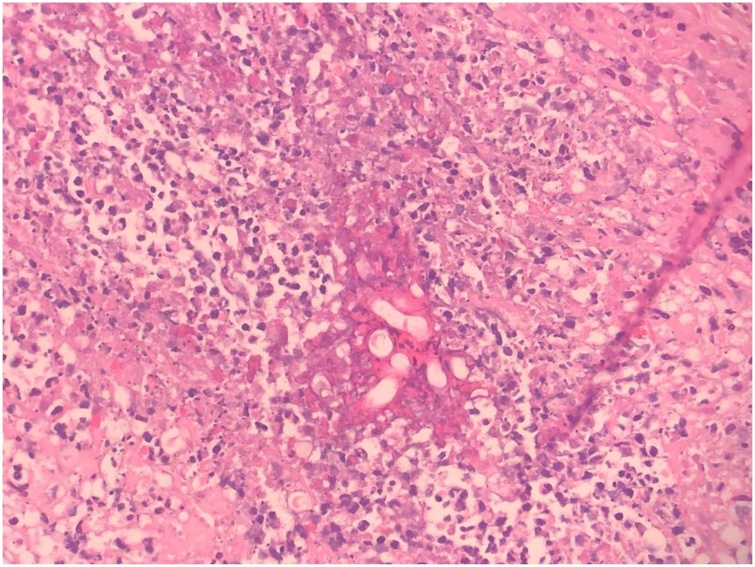
Transverse and longitudinal sections of the mass showing irregular wide hyphae with occasional septa.

**Fig. 6:**
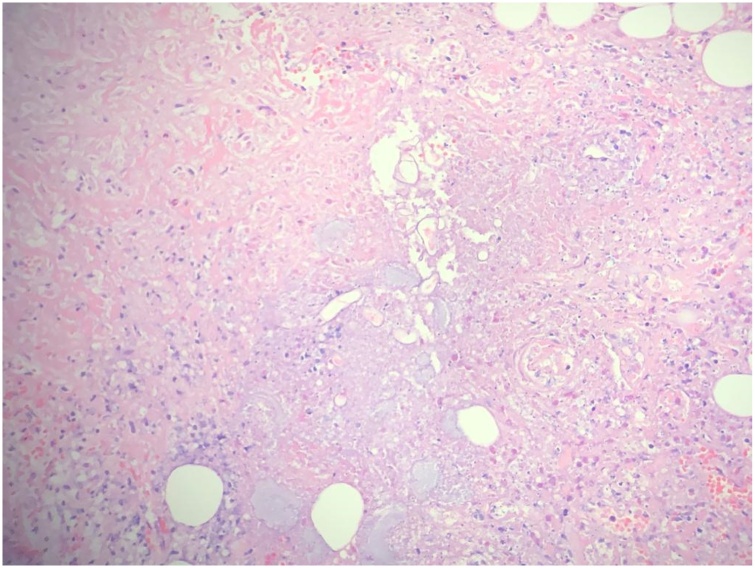
Transverse and longitudinal sections of the mass showing fungal hyphae, the sunburst pattern of Splendore–Hoeppli phenomenon, and numerous eosinophils.

Two weeks after the patient’s death, the cytology results came back negative for malignancy. Histopathology was negative for malignancy, but was consistent with intestinal basidiobolomycosis. The typical picture comprised zygospores and irregular wide hyphae with occasional septa, surrounded by thick eosinophilic material (Splendore-Hoeppli phenomenon; Fig. 5, Fig. 6). A mixed inflammatory cell infiltrate (eosinophils, neutrophils, and few giant cells) was present in the submucosa and pericolic fat. Fungal invasion of blood vessels was evident.

## 3 Discussion

Gastrointestinal basidiobolomycosis is a rare fungal infection caused by *Basidiobolus ranarum.* It can affect major organs such as the stomach, small intestines, colon, and liver [1,2]. Because the patient presents with nonspecific signs and symptoms [3], the diagnosis is easily missed or delayed. Timely, accurate diagnosis is crucially important, especially when there are features of obstruction and sepsis.

Our patient presented with history of abdominal pain for 2 months, but did not respond to the initial treatment. Imaging studies were suggestive of malignancy, but histopathologic analysis showed a mixed inflammatory cell infiltrate with thick eosinophilic material (Splendore–Hoeppli phenomenon). Consistent with a previous case series of patients with GIB [2], our patient had a high white blood cell count.

Persistent severe abdominal pain in a patient with neutropenia should alert the physician to the possibility of a fungal infection such as zygomycosis [4]. Diagnosis is usually by fungal staining of biopsy tissue [5]. Definitive diagnosis requires culture of the organism but, unfortunately, fungal culture is positive in only 15 %–25 % of cases [6]. Polymerase chain reaction can be helpful when fungal culture is negative. Molecular diagnostic tests are more accurate because DNA can be isolated even from formalin-fixed paraffin-embedded tissue. However, due to the rarity of GIB, the technology for molecular diagnosis is available in only a few centers [7].

Basidiobolomycosis in immunocompetent hosts has been reported from many countries: 23 reports from Saudi Arabia and the US, 17 from Iran, 2 from Kuwait, 6 from Iraq, 2 from Nigeria, 4 from Brazil, and 1 from the Netherlands [2,3]. Between 2014 and 2018, seven additional cases were reported: 5 from Saudi Arabia, 1 from the US, and 1 from India [8]. GIB does not show a predilection for any particular age-group and has been reported in patients aged 1.5 years to 80 years [9]. Currently available data suggests that males may be more susceptible than females; only 6 of the 71 reported cases were in females [3]. The risk for basidiobolomycosis may be higher in individuals with uncontrolled diabetes mellitus (particularly with ketoacidosis), prolonged neutropenia, prolonged corticosteroid use, hematological malignancy, organ transplant, iron overload, acquired immunodeficiency syndrome (AIDS), injection drug use, trauma/burns, and malnutrition [1,6]. Our patient did not have any of these risk factors. Although she had a history of *H. pylori* infection, there was evidence that she had received ranitidine, which may increase susceptibility to GIB by suppressing acid production [10]. In a case series of 20 patients with gastrointestinal mucormycosis, Thomson et al. found that the stomach was the organ most commonly affected, and patients usually presented with the symptoms of a peptic ulcer. They reported that, in the presence of histopathological evidence of fungal vascular invasion, the disease was usually fatal [11]. Our patient had histopathological evidence of fungal vascular invasion.

The limited data available suggests that treatment of mucormycosis is with surgical debridement of infected tissues and antifungal therapy [12]; this was also the treatment that we used for our patient. According to a previous report, without surgical intervention, this fungal infection is invariably fatal; antifungal treatment alone may not be enough [13]. However, surgery may not be practicable in patients with infection at multiple sites or low platelet and neutrophil counts, when the prognosis is usually grim [13]. During treatment of basidiobolomycosis it is also important to address factors such as metabolic acidosis, neutropenia, hyperglycemia, iron overload, and use of immunosuppressive drugs.

Because of the difficulty in establishing a definite diagnosis, treatment is often presumptive. Amphotericin B (preferably the lipid formulation) is preferred because of its low renal toxicity [14]. Antifungal agents such as fluconazole, itraconazole, voriconazole, and caspofungin have not shown efficacy against *Mucor* in clinical and in vitro studies [15]. Posaconazole or isavuconazole are used as step-down therapy for patients who have responded to amphotericin B. Both drugs can also be used as salvage therapy for patients who do not respond to, or cannot tolerate, amphotericin B. For salvage therapy, the choice of the oral vs. the intravenous route depends on how ill the patient is, whether an initial course of amphotericin B can be administered, and whether the patient has a functioning gastrointestinal tract [16].

In conclusion, zygomycosis is a rare cause of large bowel obstruction. Early diagnosis and treatment are crucial as the disease can be fatal. GIB in an immunocompetent host is extremely rare and so the diagnosis may be easily missed. Further research is needed to identify the factors that predispose immunocompetent individuals to GIB. This work has been reported in line with the SCARE 2018 criteria [17].

## Declaration of Competing Interest

The authors report no declarations of interest.

## Funding

This research did not receive any specific grant from funding agencies in the public, commercial, or not-for-profit sectors.

## Ethical approval

In our institute, ethical approval is exempted, depend on acquired patient consent.

## Consent

Written informed consent was obtained from the guardian on behalf of the patient for publication of this case report and accompanying images. A copy of the written consent is available for review by the Editor-in-Chief of this journal on requested.

## Author contribution

All authors contributed to manuscript preparation, manuscript editing, manuscript review.

## Registration of research studies

Not applicable.

## Guarantor

The Guarantor is DR. Alaa aljohani.

## Provenance and peer review

Not commissioned, externally peer-reviewed.
